# Kidney function, albuminuria and life expectancy

**DOI:** 10.1186/s40697-014-0033-6

**Published:** 2014-12-19

**Authors:** Tanvir Chowdhury Turin, Sofia B Ahmed, Marcello Tonelli, Braden Manns, Pietro Ravani, Matthew James, Robert R Quinn, Min Jun, Ron Gansevoort, Brenda Hemmelgarn

**Affiliations:** Department of Family Medicine, University of Calgary, Room G012F, 3330 Hospital Drive Northwest, Calgary, Alberta T2N 4N1 Canada; Department of Community Health Sciences, University of Calgary, Calgary, Alberta Canada; Libin Cardiovascular Institute, University of Calgary, Calgary, Alberta Canada; Institute of Public Health, University of Calgary, Calgary, Alberta Canada; Department of Medicine, University of Calgary, Calgary, Alberta Canada; Department of Medicine, University of Alberta, Edmonton, Alberta Canada; Department of Nephrology, University Medical Center Groningen, Groningen, The Netherlands

## Abstract

**Background:**

Lower estimated glomerular filtration rate is associated with reduced life expectancy. Whether this association is modified by the presence or absence of albuminuria, another cardinal finding of chronic kidney disease, is unknown.

**Objective:**

Our objective was to estimate the life expectancy of middle-aged men and women with varying levels of eGFR and concomitant albuminuria.

**Design:**

A retrospective cohort study.

**Setting:**

A large population-based cohort identified from the provincial laboratory registry in Alberta, Canada.

**Participants:**

Adults aged ≥30 years who had outpatient measures of serum creatinine and albuminuria between May 1, 2002 and March 31, 2008.

**Measurements:**

*Predictor*: Baseline levels of kidney function identified from serum creatinine and albuminuria measurements. *Outcomes*: all cause mortality during the follow-up.

**Methods:**

Patients were categorized based on their estimated glomerular filtration rate (eGFR) (≥60, 45–59, 30–44, and 15–29 mL/min/1 · 73 m^2^) as well as albuminuria (normal, mild, and heavy) measured by albumin-to-creatinine ratio or urine dipstick. The abridged life table method was applied to calculate the life expectancies of men and women from age 40 to 80 years across combined eGFR and albuminuria categories. We also categorized participants by severity of kidney disease (low risk, moderately increased risk, high risk, and very high risk) using the combination of eGFR and albuminuria levels.

**Results:**

Among men aged 50 years and with eGFR ≥60 mL/min/1.73 m^2^, estimated life expectancy was 24.8 (95% CI: 24.6-25.0), 17.5 (95% CI: 17.1-17.9), and 13.5 (95% CI: 12.6-14.3) years for participants with normal, mild and heavy albuminuria respectively. Life expectancy for men with mild and heavy albuminuria was 7.3 (95% CI: 6.9-7.8) and 11.3 (95% CI: 10.5-12.2) years shorter than men with normal proteinuria, respectively. A reduction in life expectancy was associated with an increasing severity of kidney disease; 24.8 years for low risk (95% CI: 24.6-25.0), 19.1 years for moderately increased risk (95% CI: 18.7-19.5), 14.2 years for high risk (95% CI: 13.5-15.0), and 9.6 years for very high risk (95% CI: 8.4-10.8). Among women of similar age and kidney function, estimated life expectancy was 28.9 (95% CI: 28.7-29.1), 19.8 (95% CI: 19.2-20.3), and 14.8 (95% CI: 13.5-16.0) years for participants with normal, mild and heavy albuminuria respectively. Life expectancy for women with mild and heavy albuminuria was 9.1 (95% CI: 8.5-9.7) and 14.2 (95% CI: 12.9-15.4) years shorter than the women with normal proteinuria, respectively. For women also a graded reduction in life expectancy was observed across the increasing severity of kidney disease; 28.9 years for low risk (95% CI: 28.7-29.1), 22.5 years for moderately increased risk (95% CI: 22.0-22.9), 16.5 years for high risk (95% CI: 15.4-17.5), and 9.2 years for very high risk (95% CI: 7.8-10.7).

**Limitations:**

Possible misclassification of long-term kidney function categories cannot be eliminated. Possibility of confounding due to concomitant comorbidities cannot be ruled out.

**Conclusion:**

The presence and degree of albuminuria was associated with lower estimated life expectancy for both gender and was especially notable in those with eGFR ≥30 mL/min/1.73 m^2^. Life expectancy associated with a given level of eGFR differs substantially based on the presence and severity of albuminuria.

**Electronic supplementary material:**

The online version of this article (doi:10.1186/s40697-014-0033-6) contains supplementary material, which is available to authorized users.

## Abrégé

**Contexte:**

La diminution du débit de la filtration glomérulaire est associée à la réduction de l’espérance de vie. Par contre, le rôle que joue un autre facteur important de la néphropathie chronique dans ce lien, soit la présence ou l’absence d’albuminurie, reste inconnu.

**Objectifs:**

Notre objectif était d’estimer l’espérance de vie d’hommes et de femmes d’âge moyen avec des mesures du débit de filtration glomérulaire estimé (eDFG) et de dosages d’albuminuries concomitantes.

**Type d’étude:**

Étude rétrospective de cohortes.

**Contexte:**

Une vaste cohorte de population provenant du registre du laboratoire provincial de l’Alberta, au Canada

**Participants:**

Adultes âgés ≥ 30 ans ayant fait mesurer leurs concentrations de créatinine sérique et d’albumine urinaire en clinique externe entre le 1er mai 2002 et le 31 mars 2008.

**Mesures:**

Variable prédictive: État de la fonction rénale à l’entrée de l’étude, telle qu’évaluée en fonction des dosages de créatinémie et d’albuminurie. Résultat: toute mortalité, quelle qu’en soit la cause, pendant le suivi.

**Méthodes:**

Les patients ont été classés en fonction de leur évaluation du débit de filtration glomérulaire estimé (eDFG) (60, 45–59, 30–44, et 15–29 mL/min/1,73 m^2^) et de leur concentration d’albumine urinaire (normale, basse, élevée): mesures prises soit par rapport albumine/créatinine, ou par analyse au moyen d’une bandelette réactive. La méthodologie des tables abrégées de mortalité a été utilisée pour calculer l’espérance de vie des hommes et des femmes âgés de 40 à 80 ans à l’intérieur des deux catégories combinées. Nous avons également catégorisé les participants par degré de sévérité de néphropathie (faible risque, risques modéré et élevé, et très haut risque) en combinant les résultats des évaluations du eDFG et des dosages d’albuminurie.

**Résultats:**

Parmi les hommes âgés de 50 ans qui avaient un eDFG ≥60 mL/min/1,73 m2, l’espérance de vie était estimée à 24,8 (95% CI: 24,6-25,0), 17,5 (95% CI: 17,1-17,9), et 13,5 (95% CI: 12,6-14,3) années pour les participants ayant des concentrations d’albuminurie normales, basses et élevées, respectivement. L’espérance de vie pour les hommes avec des concentrations d’albuminurie basses et élevées était écourtée de 7,3 (95% CI: 6,9-7,8) et de 11,3 (95% CI: 10,5-12,2) années, comparativement aux hommes ayant obtenu des concentrations de protéinurie normales. Une diminution de l’espérance de vie a été associée avec le degré de sévérité de la néphropathie: 24,8 ans pour un risque faible (95% CI: 24,6-25,0), 19,1 ans pour un risque modéré (95% CI: 18,7-19,5), 14,2 ans pour un risque élevé (95% CI: 13,5-15,0), et 9,6 ans pour un très haut risque (95% CI: 8,4-10,8). Parmi les femmes d’âge et d’état de la fonction rénale similaires, l’espérance de vie était estimée à 28,9 (95% CI: 28,7-29,1), 19,8 (95% CI: 19,2-20,3), et 14,8 (95% CI: 13,5-16,0) années pour les participantes ayant des concentrations d’albumine urinaire normales, basses et élevées, respectivement. L’espérance de vie pour les femmes avec des concentrations d’albuminurie basses et élevées était écourtée de 9,1 (95% CI: 8,5-9,7) et de 14,2 (95% CI: 12,9-15,4) années comparativement aux femmes obtenant des concentrations normales de protéinurie, respectivement. Pour les femmes, également, l’espérance de vie diminuait au fur et à mesure que le degré de sévérité de la néphropathie augmentait: de 28,9 ans pour celles à faible risque (95% CI: 28,7-29,1), de 22,5 ans pour celles à risque modéré (95% CI: 22,0-22,9), de 16,5 ans pour celles à risque élevé (95% CI: 15,4-17,5), et de 9,2 ans pour celles à très haut risque (95% CI: 7,8-10,7).

**Limites de l’étude:**

Nous ne pouvons éliminer la possibilité d’une erreur lors du classement à partir de l’état de la fonction rénale. Les facteurs de confusion causés par une comorbidité concomitante ne peuvent, également, être complètement éliminés.

**Conclusion:**

La présence et la sévérité de l’albuminurie ont été associées avec une espérance de vie diminuée chez les participants des deux sexes et était particulièrement appréciable chez les sujets avec un DFG de ≥30 mL/min/1.73 m2. L’espérance de vie associée à un DFG donné variait considérablement selon la présence d’albuminurie et sa sévérité.

## What was known before

A lower level of eGFR is associated with shorter life span as is the presence and severity of albuminuria. Despite its obvious implications for clinical practice, public policy, and communicating risk to members of the public the joint effects of eGFR and albuminuria on estimated life expectancy have not been reported.

## What this adds

The life expectancy estimates presented provide a useful summary of risk based on combined eGFR and albuminuria levels. The results indicate that life expectancy varies substantially based on level of eGFR and presence and severity of albuminuria.

## Background

Until recently, the presence of chronic kidney disease (CKD) was primarily identified by estimated glomerular filtration rate (eGFR). Recent guidelines [1] for CKD classification and management recommend that degree of albuminuria also be used when classifying the presence and severity of CKD, because (like lower GFR) more severe albuminuria is associated with an increased risk of death [2-4].

Life expectancy is an alternative measure that can be used to estimate health status and disease burden at the population level. Life expectancy is typically reported as the estimated remaining years of life, and may be more readily understood by the general population than other measures such as relative risk and hazard ratio. A lower level of eGFR is associated with shorter life expectancy [5], as is the presence and severity of albuminuria [6]. Despite its obvious implications for clinical practice, public policy, and communicating risk to members of the public the joint effects of eGFR and albuminuria on estimated life expectancy have not been reported.

We used a large population-based cohort in a single Canadian province to estimate the life expectancy of middle-aged men and women with varying levels of eGFR and concomitant albuminuria.

## Methods

### Data source

The study population included adults 30 years of age and older in Alberta, Canada who had at least 1 outpatient measure of eGFR and at least outpatient 1 measure of albuminuria between May 1, 2002 and March 31, 2008 [7]. Patients were excluded if they were treated with dialysis or a kidney transplant at study entry or had baseline eGFR <15 mL/min per 1 · 73 m^2^. Participants were followed until March 31, 2009 to identify all-cause mortality, determined from Vital Statistics data of the Alberta Health Registry file [7]. Ethics approval for this study was obtained from the Institutional Review Board of the University of Calgary.

### Measurement of kidney function and albuminuria

We used the date of the first serum creatinine measurement as the index date and the CKD-EPI equation [8] was used to estimate the eGFR for each patient. Baseline kidney function (index eGFR) was categorized as ≥60, 45–59, 30–44, and 15–29 mL/min/1 · 73 m^2^ [5,9]. Baseline albuminuria was determined by urine albumin:creatinine ratio (ACR) or urine dipstick based on outpatient random spot urine measurements in the 6-month periods before and after the index eGFR. If a patient had both ACR and urine dipstick measurement, the ACR was utilized. For patients with multiple measurements, the median of all respective measurements was selected as the baseline. Albuminuria was categorized as normal, mild, or heavy based on ACR (normal: <30 mg/g; mild: 30–300 mg/g, or heavy: >300 mg/g) or urine dipstick (normal: negative; mild: trace or 1+, or heavy: 2+) [6,10].

In the primary analysis, we present all combinations of eGFR and albuminuria. In a secondary analysis, we present combinations of eGFR and albuminuria according to categories of CKD severity defined by the KDIGO 2012 Clinical Practice Guideline [1]: low risk, moderate risk, high risk, and very high risk.

### Statistical analysis

We performed stratified analysis by sex as prior literature indicates that the mortality implications of CKD may differ for men and women [11]. Age was considered in the time scale and age bands used in the life expectancy estimates were set at five years, beginning at age 40 year with the highest age category set at age 80 years and older. Age-specific mortality rates were calculated with the person-year method [12]. The abridged life table was used to calculate life expectancies from these age-group specific mortality rates [13,14]. Abridged life tables and associated variances, standard errors, and 95% confidence intervals (CI) were calculated according to Chiang’s method [15]. Differences in life expectancy, as well as the 95% CI, between kidney function categories were estimated.

### Sensitivity analysis

Analyses were repeated among a subset of the population after excluding participants with history of diabetes, hypertension, and cardiovascular diseases (including acute myocardial infarction, congestive heart failure, and stroke).

## Results

**Figure 1:**
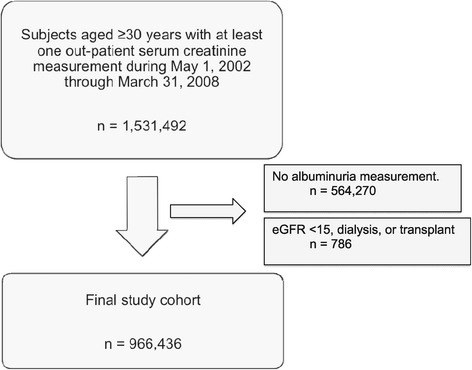
**Cohort formation.**

During the study period 966,436 subjects (453,257 men and 513,179 women) had at least one outpatient serum creatinine measurement and one albuminuria measurement (Figure 1). The majority of male participants had eGFR ≥60 ml/min/1.73 m^2^ range: 93.1% had eGFR ≥60, 5.0% had eGFR 45–59, 1.5% had eGFR 30–44,and 0.5% had eGFR 15–29 ml/min/1.73 m^2^. The corresponding proportions among female participants were 91.6%, 5.8%, 2.0%, and 0.6%, respectively. Among male participants, 89.2% had no albuminuria, 9.0% had mild albuminuria, and 1.8% had heavy albuminuria, while among female participants the corresponding proportions were 92.1%, 6.8% and 1.1%, respectively. Table 1 shows demographic and clinical characteristics of participants, by level of kidney function or albuminuria.

**Table 1:** **Demographic and clinical characteristics of participants, by level of kidney function or proteinuria**

	**eGFR (mL/min/1.73 m** ^**2**^ **)**				**Albuminuria**		
	**N = 966,436**				**N = 966,436**		
	**≥60**	**45-59**	**30-44**	**15-29**	**None**	**Mild**	**Heavy**
	**N = 892,350**	**N = 52,237**	**N = 16,926**	**N = 4,923**	**N = 877,076**	**N = 75,421**	**N = 13,939**
**Female**	53	58	61	59	60	51	42
**Aboriginal**	1	1	1	2	2	3	4
**Diabetes,**	12	21	29	35	9	18	34
**Hypertension**	38	65	81	84	29	41	56
**Cerebrovascular dis**	3	7	11	12	2	4	7
**Congestive heart failure**	2	8	15	25	2	5	9
**Myocardial infarction**	3	6	11	15	2	4	6
**Socioeconomic status**							
**Pensioner**	33	73	84	78	22	31	35
**Low**	18	12	7	8	15	14	14
**Low with subsidy**	2	2	2	3	3	5	6

Data expressed as; eGFR estimated glomerular filtration rate; Socioeconomic status was categorized as high (annual adjusted taxable family income ≥ $39 250 CAD), low (annual adjusted taxable family income < $39 250 CAD), low with subsidy (receiving social assistance), and pensioners (age 65 years and older) based on government of Alberta health care insurance records.

Tables 2 and 3 show estimated life expectancy among male and female participants respectively across eGFR categories by level of albuminuria for 5-year age bands (from 40 years of age to 80 years). Among men aged 50 years and with eGFR ≥60 mL/min/1.73 m^2^, as shown in Table 2, estimated life expectancy was 24.8 years for participants without albuminuria, 17.5 years for participants with mild albuminuria, and 13.5 years for participants with heavy albuminuria. Table 3 shows estimated life expectancy among female participants across eGFR categories by level of albuminuria. Among women of the same age and eGFR category, estimated life expectancy was 28.9 years for participants without albuminuria, 19.8 years for participants with mild albuminuria, and 14.8 years for participants with heavy albuminuria.

**Table 2:** **Life expectancy (95% confidence interval) for men, across eGFR categories by albuminuria levels**

**eGFR category**	**Age group (year)**	**Albuminuria**		
		**Normal**	**Mild**	**Heavy**
≥60	40	33.4 (33.2 to 33.6)	24.8 (24.2 to 25.3)	19.1 (17.8 to 20.3)
	45	29.1 (28.9 to 29.2)	20.9 (20.4 to 21.3)	16.1 (15.1 to 17.1)
	50	24.8 (24.6 to 25.0)	17.5 (17.1 to 17.9)	13.5 (12.6 to 14.3)
	55	20.8 (20.7 to 21.0)	14.6 (14.2 to 14.9)	10.5 (9.8 to 11.3)
	60	17.1 (16.9 to 17.3)	11.8 (11.4 to 12.1)	8.6 (7.9 to 9.3)
	65	13.5 (13.4 to 13.7)	9.3 (9.0 to 9.7)	6.9 (6.3 to 7.4)
	70	10.4 (10.2 to 10.5)	7.2 (6.9 to 7.4)	5.2 (4.7 to 5.6)
	75	7.7 (7.6 to 7.9)	5.4 (5.2 to 5.6)	4.4 (4.0 to 4.8)
	80	5.5 (5.3 to 5.7)	3.9 (3.7 to 4.1)	3.3 (2.9 to 3.6)
45-59	40	30.4 (28.7 to 32.2)	21.1 (17.5 to 24.7)	17.2 (13.8 to 20.6)
	45	26.5 (25.2 to 27.8)	18.3 (16.1 to 20.6)	13.3 (10.4 to 16.2)
	50	23.1 (22.2 to 24.1)	14.9 (13.1 to 16.6)	10.8 (8.6 to 13.1)
	55	19.7 (19.0 to 20.4)	11.9 (10.5 to 13.2)	10.3 (8.8 to 11.8)
	60	16.3 (15.8 to 16.8)	10.1 (9.1 to 11.1)	7.2 (6.0 to 8.4)
	65	13.0 (12.6 to 13.4)	8.6 (7.9 to 9.2)	6.4 (5.5 to 7.3)
	70	10.1 (9.8 to 10.4)	6.7 (6.2 to 7.2)	4.9 (4.3 to 5.6)
	75	7.5 (7.2 to 7.7)	4.9 (4.5 to 5.2)	3.6 (3.2 to 4.0)
	80	5.1 (4.9 to 5.3)	3.5 (3.3 to 3.8)	2.6 (2.0 to 3.2)
30-44	40	16.8 (11.2 to 22.3)	15.2 (7.7 to 22.7)	15.2 (10.8 to 19.6)
	45	13.5 (8.4 to 18.6)	16.0 (11.0 to 21.1)	12.2 (9.4 to 15.1)
	50	15.5 (12.6 to 18.3)	14.7 (11.8 to 17.5)	8.2 (5.8 to 10.6)
	55	12.6 (10.5 to 14.6)	10.7 (8.5 to 13.0)	7.0 (5.3 to 8.6)
	60	10.9 (9.6 to 12.3)	8.7 (7.4 to 10.0)	5.2 (4.1 to 6.4)
	65	8.9 (8.1 to 9.8)	6.0 (5.1 to 6.9)	5.0 (4.1 to 6.0)
	70	7.2 (6.6 to 7.7)	5.0 (4.4 to 5.6)	4.9 (4.2 to 5.6)
	75	5.6 (5.2 to 5.9)	4.1 (3.7 to 4.5)	3.3 (2.9 to 3.7)
	80	3.7 (3.3 to 4.0)	3.1 (2.9 to 3.3)	2.7 (2.5 to 2.9)
15-29	40	6.6 (1.2 to 12.1)	8.6 (2.9 to 14.2)	8.3 (6.7 to 14.4)
	45	6.8 (0.9 to 12.7)	8.6 (4.6 to 12.7)	6.7 (5.5 to 11.1)
	50	12.6 (6.8 to 18.5)	6.5 (3.1 to 10.0)	6.9 (4.4 to 9.1)
	55	10.5 (6.7 to 14.4)	8.3 (5.4 to 11.1)	6.1 (4.9 to 8.9)
	60	8.4 (6.1 to 10.7)	5.3 (3.3 to 7.3)	4.5 (4.6 to 7.6)
	65	6.0 (4.6 to 7.3)	5.5 (3.9 to 7.1)	3.9 (3.6 to 5.4)
	70	4.5 (3.6 to 5.3)	4.7 (3.8 to 5.6)	2.7 (2.5 to 3.2)
	75	3.7 (3.2 to 4.3)	3.0 (2.6 to 3.3)	1.9 (2.5 to 3.0)
	80	2.8 (2.6 to 3.0)	2.4 (1.9 to 3.0)	1.3 (1.3 to 2.6)

**Table 3:** **Life expectancy (95% confidence interval) for women, across eGFR categories by albuminuria levels**

**eGFR category**	**Age group (year)**	**Albuminuria**		
		**Normal**	**Mild**	**Heavy**
≥60	40	37.8 (37.6 to 38.0)	27.2 (26.6 to 27.9)	22.1 (20.5 to 23.6)
	45	33.3 (33.1 to 33.5)	23.4 (22.8 to 24.0)	18.5 (17.2 to 19.9)
	50	28.9 (28.7 to 29.1)	19.8 (19.2 to 20.3)	14.8 (13.5 to 16.0)
	55	24.6 (24.5 to 24.8)	16.6 (16.1 to 17.1)	12.0 (10.9 to 13.1)
	60	20.6 (20.4 to 20.8)	13.4 (13.0 to 13.9)	9.2 (8.2 to 10.2)
	65	16.8 (16.6 to 17.0)	10.6 (10.2 to 11.1)	7.7 (6.9 to 8.6)
	70	13.1 (13.0 to 13.3)	8.3 (7.9 to 8.6)	5.7 (5.0 to 6.4)
	75	9.9 (9.7 to 10.0)	6.4 (6.1 to 6.7)	4.7 (4.1 to 5.2)
	80	6.9 (6.7 to 7.1)	4.5 (4.2 to 4.8)	3.2 (2.9 to 3.6)
45-59	40	32.8 (31.1 to 34.5)	23.6 (18.9 to 28.3)	18.9 (13.7 to 24.1)
	45	29.3 (28.2 to 30.4)	18.6 (13.9 to 23.3)	16.3 (13.0 to 19.5)
	50	25.5 (24.6 to 26.3)	18.3 (15.2 to 21.4)	11.3 (8.0 to 14.5)
	55	21.7 (21.1 to 22.4)	15.3 (12.9 to 17.8)	10.2 (7.9 to 12.5)
	60	18.6 (18.1 to 19.1)	13.7 (11.9 to 15.5)	8.0 (6.2 to 9.8)
	65	15.1 (14.7 to 15.5)	12.8 (11.6 to 13.9)	7.1 (5.8 to 8.4)
	70	12.0 (11.7 to 12.3)	9.4 (8.6 to 10.3)	5.3 (4.4 to 6.3)
	75	9.1 (8.9 to 9.3)	7.6 (7.0 to 8.2)	4.3 (3.6 to 4.9)
	80	6.3 (6.1 to 6.5)	6.3 (5.9 to 6.8)	2.9 (2.2 to 3.7)
30-44	40	26.7 (23.1 to 30.3)	14.0 (8.1 to 19.9)	11.6 (6.7 to 16.5)
	45	21.7 (18.1 to 25.3)	11.6 (7.0 to 16.2)	9.8 (5.7 to 14.0)
	50	18.9 (16.6 to 21.2)	10.5 (6.7 to 14.4)	10.7 (7.6 to 13.7)
	55	15.4 (13.7 to 17.2)	10.0 (7.6 to 12.5)	7.7 (5.5 to 9.9)
	60	13.7 (12.4 to 14.9)	8.9 (7.2 to 10.6)	6.0 (4.3 to 7.7)
	65	11.6 (10.7 to 12.4)	7.1 (6.0 to 8.2)	5.2 (3.9 to 6.5)
	70	9.7 (9.2 to 10.2)	4.9 (4.2 to 5.5)	5.1 (4.1 to 6.1)
	75	7.2 (6.8 to 7.5)	4.7 (4.2 to 5.2)	3.5 (2.9 to 4.0)
	80	4.8 (4.6 to 5.1)	3.8 (3.5 to 4.1)	3.2 (2.8 to 3.5)
15-29	40	15.0 (4.3 to 25.7)	14.6 (10.7 to 18.5)	6.2 (2.1 to 10.3)
	45	16.5 (12.7 to 20.4)	9.6 (5.7 to 13.5)	5.3 (2.5 to 8.1)
	50	11.5 (7.7 to 15.4)	6.2 (2.9 to 9.5)	5.5 (2.9 to 8.0)
	55	8.0 (4.8 to 11.3)	7.8 (5.4 to 10.1)	6.1 (4.1 to 8.1)
	60	10.4 (8.6 to 12.2)	5.4 (3.8 to 7.0)	5.3 (3.8 to 6.8)
	65	6.8 (5.6 to 8.1)	3.6 (2.7 to 4.6)	3.6 (2.7 to 4.6)
	70	4.9 (4.1 to 5.7)	3.3 (2.7 to 3.9)	3.1 (2.5 to 3.7)
	75	5.1 (4.5 to 5.6)	3.5 (2.9 to 4.1)	2.8 (2.4 to 3.2)
	80	3.7 (3.4 to 4.0)	2.6 (2.5 to 2.8)	1.8 (1.2 to 2.4)

Tables 4 and 5 show the difference in estimated life expectancy between normal versus mild albuminuria, and between normal versus severe albuminuria for both men and women participants, by eGFR category. Within each eGFR category, estimated life expectancy was shorter for people with higher levels of albuminuria. The estimated life expectancy of 50-year old men with eGFR ≥60 and no albuminuria was 7.3 and 11.3 years longer than those with similar eGFR but with mild and heavy albuminuria, respectively. For women of same age, the estimated life expectancy for eGFR ≥60 and no albuminuria was 9.1 and 14.2 years longer than those with similar eGFR but with mild and heavy albuminuria, respectively. In general, estimated life expectancy was shorter in the presence of mild and heavy albuminuria (compared to those with no albuminuria) for both males and females.

**Table 4:** **Difference in life expectancy (95% confidence interval) by albuminuria levels, across the eGFR category among men**

**eGFR category**	**Age group (year)**	**Difference in life expectancy (95% CI)**	
		**Normal Vs mild**	**Normal Vs heavy**
eGFR ≥60	40	8.7 (8.1 to 9.2)	14.3 (13.1 to 15.6)
	45	8.2 (7.7 to 8.7)	13.0 (11.9 to 14.0)
	50	7.3 (6.9 to 7.8)	11.3 (10.5 to 12.2)
	55	6.3 (5.9 to 6.7)	10.3 (9.6 to 11.1)
	60	5.3 (4.9 to 5.7)	8.5 (7.8 to 9.2)
	65	4.2 (3.8 to 4.5)	6.7 (6.1 to 7.3)
	70	3.2 (2.9 to 3.5)	5.2 (4.7 to 5.7)
	75	2.3 (2.1 to 2.6)	3.4 (2.9 to 3.8)
	80	1.6 (1.3 to 1.8)	2.2 (1.9 to 2.6)
eGFR 45-59	40	9.3 (5.3 to 13.3)	13.2 (9.4 to 17.1)
	45	8.1 (5.5 to 10.8)	13.2 (10.0 to 16.3)
	50	8.2 (6.3 to 10.2)	12.3 (9.8 to 14.7)
	55	7.8 (6.3 to 9.4)	9.4 (7.7 to 11.0)
	60	6.2 (5.1 to 7.4)	9.1 (7.8 to 10.4)
	65	4.5 (3.7 to 5.3)	6.6 (5.6 to 7.6)
	70	3.4 (2.8 to 3.9)	5.1 (4.4 to 5.8)
	75	2.6 (2.2 to 3.0)	3.9 (3.4 to 4.4)
	80	1.6 (1.3 to 1.9)	2.5 (1.9 to 3.1)
eGFR 30-44	40	1.6 (−7.8 to 10.9)	1.6 (−5.5 to 8.7)
	45	−2.5 (−9.7 to 4.6)	1.3 (−4.6 to 7.1)
	50	0.8 (−3.3 to 4.8)	7.2 (3.5 to 10.9)
	55	1.8 (−1.2 to 4.9)	5.6 (3.0 to 8.3)
	60	2.2 (0.3 to 4.1)	5.7 (3.9 to 7.5)
	65	3.0 (1.7 to 4.2)	3.9 (2.7 to 5.2)
	70	2.2 (1.4 to 3.0)	2.3 (1.4 to 3.2)
	75	1.5 (0.9 to 2.0)	2.2 (1.7 to 2.8)
	80	0.6 (0.2 to 1.0)	1.0 (0.6 to 1.3)
eGFR 15-29	40	−1.9 (−9.8 to 5.9)	−1.7 (−10.6 to 2.8)
	45	−1.8 (−9.0 to 5.4)	0.1 (−8.0 to 5.1)
	50	6.1 (−0.7 to 12.9)	5.9 (−0.4 to 12.2)
	55	2.3 (−2.5 to 7.0)	3.6 (−0.7 to 7.9)
	60	3.1 (0.0 to 6.1)	2.3 (−0.5 to 5.0)
	65	0.4 (−1.6 to 2.5)	1.5 (−0.1 to 3.1)
	70	−0.3 (−1.5 to 0.9)	1.6 (0.7 to 2.5)
	75	0.7 (0.0 to 1.3)	0.9 (0.3 to 1.5)
	80	0.4 (−0.9 to 0.9)	1.5 (−0.3 to 1.9)

**Table 5:** **Difference in life expectancy (95% confidence interval) by albuminuria levels, across the eGFR category among women**

**eGFR category**	**Age group (year)**	**Difference in life expectancy (95% CI)**	
		**Normal Vs mild**	**Normal Vs heavy**
eGFR ≥60	40	10.6 (9.9 to 11.3)	15.8 (14.2 to 17.3)
	45	9.9 (9.2 to 10.5)	14.8 (13.4 to 16.1)
	50	9.1 (8.5 to 9.7)	14.2 (12.9 to 15.4)
	55	8.0 (7.5 to 8.6)	12.7 (11.5 to 13.8)
	60	7.2 (6.6 to 7.7)	11.4 (10.3 to 12.4)
	65	6.2 (5.7 to 6.6)	9.1(8.2 to 9.9)
	70	4.9 (4.5 to 5.3)	7.5 (6.7 to 8.2)
	75	3.4 (3.1 to 3.8)	5.2 (4.6 to 5.8)
	80	2.4 (2.1 to 2.7)	3.6 (3.2 to 4.0)
eGFR 45-59	40	14.2 (9.2 to 19.1)	13.9 (8.4 to 19.4)
	45	11.0 (7.8 to 14.3)	13.1 (9.6 to 16.5)
	50	10.1 (7.5 to 12.7)	14.2 (10.8 to 17.6)
	55	8.0 (6.1 to 10.0)	11.5 (9.1 to 13.9)
	60	5.8 (4.6 to 7.1)	10.7 (8.8 to 12.5)
	65	5.7 (4.7 to 6.6)	8.0 (6.6 to 9.4)
	70	4.4 (3.7 to 5.1)	6.7 (5.7 to 7.7)
	75	2.8 (2.3 to 3.2)	4.8 (4.1 to 5.5)
	80	2.0 (1.6 to 2.3)	3.4 (2.6 to 4.1)
eGFR 30-44	40	12.7 (5.7 to 19.6)	15.1 (9.0 to 21.2)
	45	10.1 (4.3 to 15.9)	11.8 (6.3 to 17.4)
	50	8.4 (3.9 to 12.9)	8.3 (4.5 to 12.1)
	55	5.4 (2.4 to 8.5)	7.8 (5.0 to 10.6)
	60	4.8 (2.7 to 6.8)	7.6 (5.5 to 9.7)
	65	4.5 (3.1 to 5.8)	6.4 (4.8 to 7.9)
	70	4.8 (4.0 to 5.7)	4.6 (3.5 to 5.7)
	75	2.5 (1.9 to 3.0)	3.7 (3.1 to 4.3)
	80	1.0 (0.6 to 1.4)	1.7 (1.2 to 2.2)
eGFR 15-29	40	0.3 (−11.1 to 11.7)	8.8 (−2.7 to 20.3)
	45	6.9 (1.4 to 12.4)	11.2 (6.4 to 16.0)
	50	5.3 (0.3 to 10.4)	6.1 (1.4 to 10.7)
	55	0.3 (−3.8 to 4.3)	1.9 (−1.9 to 5.7)
	60	5.0 (2.6 to 7.4)	5.1 (2.8 to 7.5)
	65	3.2 (1.6 to 4.7)	3.2 (1.6 to 4.7)
	70	1.6 (0.6 to 2.6)	1.8 (0.8 to 2.8)
	75	1.6 (0.8 to 2.4)	2.3 (1.6 to 2.9)
	80	1.1 (0.7 to 1.4)	1.9 (1.3 to 2.6)

**Figure 2:**
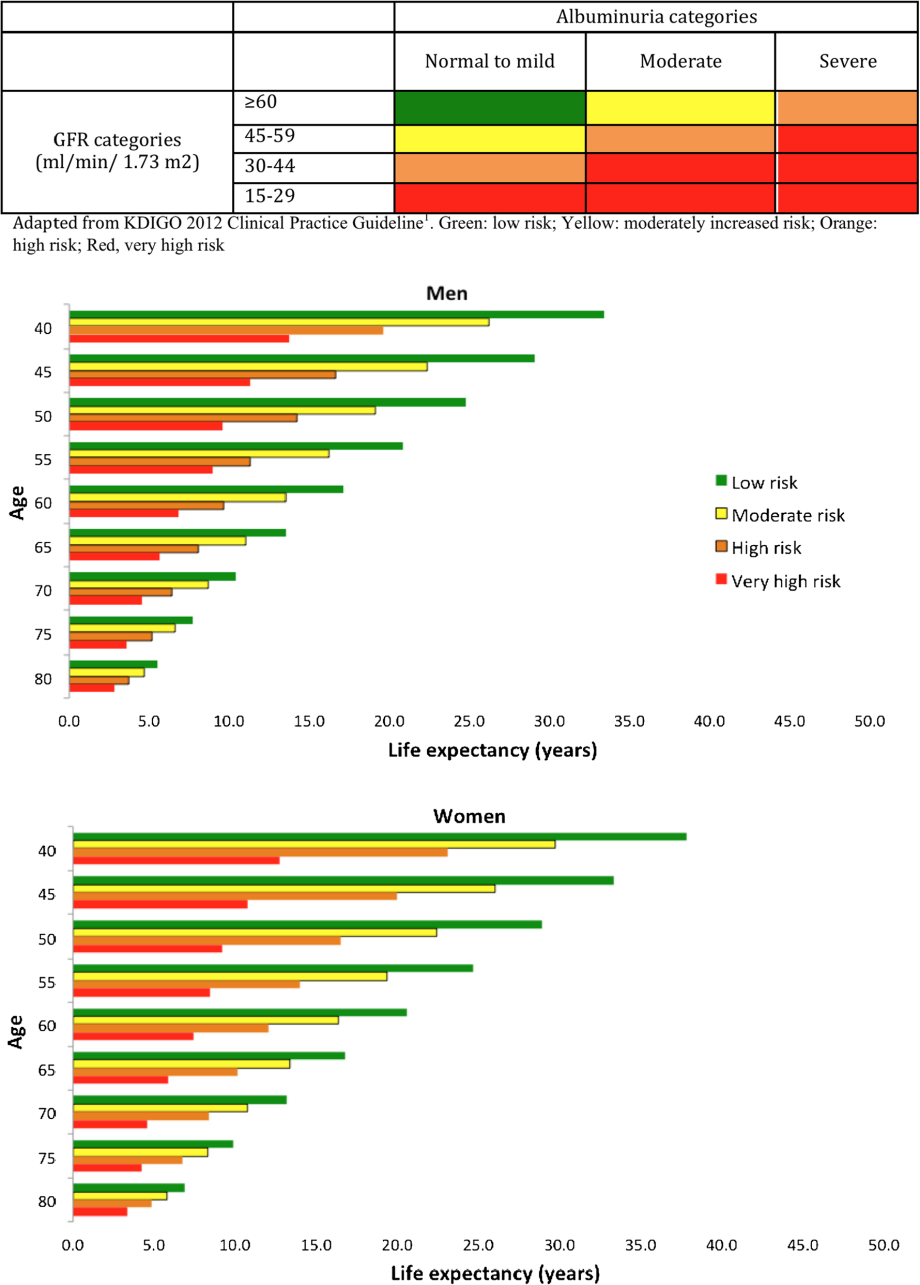
**Life expectancy among men and women at different index ages across severity of chronic kidney disease.** The heat map indicating levels of kidney disease severity is adapted from KDIGO 2012 Clinical Practice Guideline^1^. Green: low risk; Yellow: moderately increased risk; Orange: high risk; Red, very high risk.

**Figure 3:**
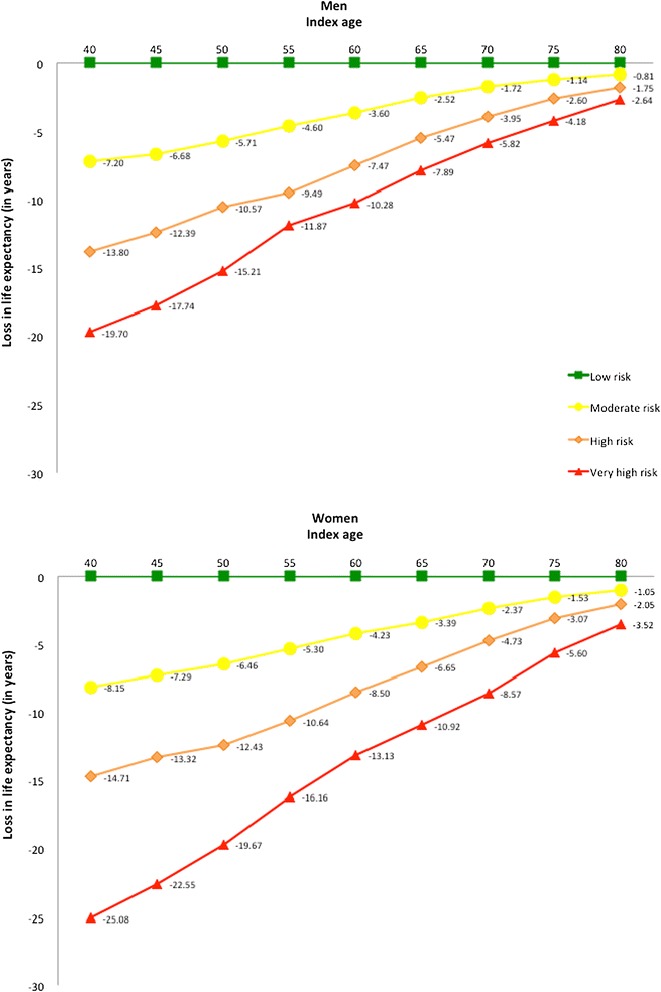
**Difference in life expectancy among men and women at different index ages across severity of chronic kidney disease.** Low risk category served as reference category. The kidney disease severity categories are adapted from KDIGO 2012 Clinical Practice Guideline^1^. Green: low risk; Yellow: moderately increased risk; Orange: high risk; Red, very high risk.

Figure 2 shows estimated life expectancy among male and female participants respectively across severity of CKD (also Additional file 1: Table S1). Among men aged 50 years, life expectancy for participants with low risk was 24.8 years, for moderately increased risk was 19.1 years, for high risk was 14.2 years, and for very high risk was 9.6 years. For women aged 50 years, life expectancy for participants with low risk was 28.9 years, for moderately increased risk was 22.5 years, for high risk was 16.5 years, and for very high risk was 9.2 years. Figure 3 shows the difference in estimated life expectancy between levels of CKD, using the “low risk” category as the referent. In reference to 50 years old men with low risk, a loss in life expectancy was 5.7 years for moderately increased risk, 10.6 years for high risk, and 15.2 years for very high risk patients. For women of 50 years age, the losses of corresponding life expectancies were 6.5, 12.4, and 19.7 years. All analyses suggested that estimated life expectancy decreased with increasing severity of CKD. Results were similar in sensitivity analyses evaluating a subset of the cohort without a history of diabetes, hypertension, or cardiovascular disease (Additional file 1: Figure S1 and S2).

## Discussion

We estimated the combined effect of kidney function and albuminuria on life expectancy for adults from a single Canadian province with a universal health care system. We observed that estimated life expectancy associated with a given level of eGFR differs substantially based on the presence and degree of albuminuria. This phenomenon was observed for both men and women, and was especially notable in those with eGFR ≥30 mL/min/1.73 m^2^. For all participants, the magnitude of the difference in estimated life expectancy associated with severe albuminuria vs mild or absent albuminuria appeared to be clinically significant. Among 40-year old men without albuminuria, individuals with CKD categories 3a, 3b and 4 have losses of 3.0, 16.6 and 26.8 years of life expectancy compared to individuals with CKD category 1 or 2. On the other hand, among 40-year old males with CKD category 1 or 2, individuals with mild albuminuria and heavy albuminuria have losses of 8.7 and 14.3 years of life expectancy compared to individuals without albuminuria. Again, among 50-year old men without albuminuria, individuals with CKD categories 3a, 3b and 4 have losses of 1.7, 9.3 and 12.2 years of life expectancy compared to individuals with CKD category 1 or 2. On the other hand, among 50-year old males with CKD category 1 or 2, individuals with mild albuminuria and heavy albuminuria have losses of 7.3 and 11.3 years of life expectancy compared to individuals without albuminuria. Among 40-year old women without albuminuria, individuals with CKD categories 3a, 3b and 4 have losses of 5.0, 11.1 and 22.8 years of life expectancy compared to individuals with CKD category 1 or 2. On the other hand, among 40-year old males with CKD category 1 or 2, individuals with mild albuminuria and heavy albuminuria have losses of 10.6 and 15.8 years of life expectancy compared to individuals without albuminuria. Among 50-year old women without albuminuria, individuals with CKD categories 3a, 3b and 4 have losses of 3.4, 10.0 and 17.4 years of life expectancy compared to individuals with CKD category 1 or 2. On the other hand, among 40-year old males with CKD category 1 or 2, individuals with mild albuminuria and heavy albuminuria have losses of 9.1 and 14.2 years of life expectancy compared to individuals without albuminuria. Even for the oldest participants (those aged 80 years), estimated life expectancy associated with eGFR 45–59.9 ml/min/1.73 m^2^ was 2.5 years (men) and 3.4 years (women) shorter when heavy albuminuria was present, as compared to when albuminuria was absent. As expected, absolute differences were more dramatic for younger participants: corresponding differences in life expectancy for women and men aged 40 years were 13.9 years and 13.2 years, respectively.

When participants were categorized according their kidney function level severity, among 40-year old men, individuals with moderate risk, high risk and very high risk have losses of 7.2, 13.8 and 19.7 years of life expectancy compared to individuals with low risk. Among 50-year old men, individuals with moderate risk, high risk and very high risk have losses of 6.7, 12.4 and 17.7 years of life expectancy compared to individuals with low risk. Among 40-year old women, individuals with moderate risk, high risk and very high risk have losses of 8.2, 14.7 and 25.1 years of life expectancy compared to individuals with low risk. Among 50-year old women, individuals with moderate risk, high risk and very high risk have losses of 7.3, 13.3 and 22.6 years of life expectancy compared to individuals with low risk.

Our findings further support the recently revised classification system for CKD [1]. Specifically, the reduction in life expectancy associated with mild or severe albuminuria was observed within each eGFR category, suggesting that albuminuria provides additional prognostic evidence beyond eGFR alone. Our observation that even mild albuminuria was associated with a clinically relevant reduction in life expectancy confirms the previous observation that a simple urine albumin assessment provides additional important prognostic information beyond serum creatinine testing alone.

Our results are especially relevant for policy-makers and for educating the public about the prognostic importance of CKD generally and albuminuria specifically. For policy-makers, our results help to clarify the population burden of CKD and its potential consequences for the general population. For the public, availability of life expectancy estimates will inform discussion about the risks associated with CKD. Although published data demonstrate that both reduced eGFR and the presence of albuminuria are associated with increased risk of death, it is uncertain whether the general population is able to make optimal use of this information. Previous work shows that estimates of life expectancy are easier for lay people to understand than measures such as incidence, prevalence, hazard ratio or relative risk [16], especially given the common lack of statistical numeracy in the general population [17]. There is also evidence to suggest patient preferences for framing health risks in absolute terms and with a lifetime perspective [18].

Previous studies have evaluated the effects of various chronic conditions on life expectancy. Loukine and colleagues reported a reduction of life expectancy of 2–3 years due to presence of hypertension among middle-aged Canadian patients [19]. Similar observations have been reported in the Finnish, Japanese, and American populations [20-22]. A substantial reduction in life expectancy was also seen among people with diabetes. Middle-aged diabetic patients from the USA, as well as Japan, had on average 7–8 years shorter life expectancy than their non-diabetic counterpart [23-25]. While hypertension and diabetes are important chronic conditions, in our study, among participants without history of hypertension, diabetes, and cardiovascular diseases, reduced kidney function also showed reduced life expectancy. This highlights the significance of CKD as a public health problem, and exemplifies the possible gain of effective primary and secondary prevention activities aimed at CKD prevention and control.

Strengths of our study include its large size, rigorous methods, and use of a population-based cohort in a setting with universal access to health care. However, the estimates reported in this study should be interpreted in the context of their limitations. Possible misclassification of long-term kidney function categories cannot be ruled out as baseline classification of kidney function level was made based on the serum creatinine measurement at one point in time with the assumption that the kidney function of individuals did not change during the follow-up period. Also due to methods applied for calculating life expectancy we were unable to adjust the estimates for concomitant comorbidities, thus the projected reductions in life expectancy associated with reduced kidney function level may be partially due to conditions such as hypertension and diabetes. The effect of this is likely to be minimal however as we observed similar results in a subset of our cohort without hypertension, diabetes or cardiovascular disease. Estimated life expectancy among the middle-aged non-CKD population in our study was approximately 7 years shorter than the overall life expectancy for men and women during the same time period in Alberta [26]. This difference is attributed to the selective nature of our study cohort, which was limited to individuals who had outpatient serum creatinine and albuminuria measurements as part of routine care and therefore does not include individuals who did not use medical services [27]. This might have resulted in inclusion of patients with comorbid conditions associated with an increased risk of adverse outcomes.

## Conclusion

In conclusion, the estimates presented here provide a useful summary of risk based on combined eGFR and albuminuria levels. The results indicate that life expectancy varies substantially based on level of eGFR and presence and severity of albuminuria. These results will aid primary care providers, researchers, and policy makers to comprehend the importance of eGFR and albuminuria combined to identify high risk groups and will be useful for in envisaging the population burden of CKD.

## Additional file

Additional file 1:
**Life expectancy among men and women at different index ages across severity of chronic kidney disease.**
