# Supplementary material for: Kidney function, albuminuria and life expectancy
Source: Can J Kidney Health Dis. 2014 Dec 19;1:33. doi: 10.1186/s40697-014-0033-6 (PMC4349777; doi:10.1186/s40697-014-0033-6)

**Supplementary Table 1: Life expectancy among men and women at different index ages across severity of chronic kidney disease*.**

**Figure Legends:**

Figure 1

Estimated life expectancy among men and women without history of diabetes, hypertension, and cardiovascular diseases at different index ages across severity of chronic kidney disease. The heat map indicating levels of kidney disease severity is adapted from KDIGO 2012 Clinical Practice Guideline. Green: low risk; Yellow: moderately increased risk; Orange: high risk; Red, very high risk.

Figure 2

Difference in estimated life expectancy among men and women without history of diabetes, hypertension, and cardiovascular diseases at different index ages across severity of chronic kidney disease. Low risk category served as reference category. The kidney disease severity categories are adapted from KDIGO 2012 Clinical Practice Guideline. Green: low risk; Yellow: moderately increased risk; Orange: high risk; Red, very high risk.

**Supplementary Figure 1**


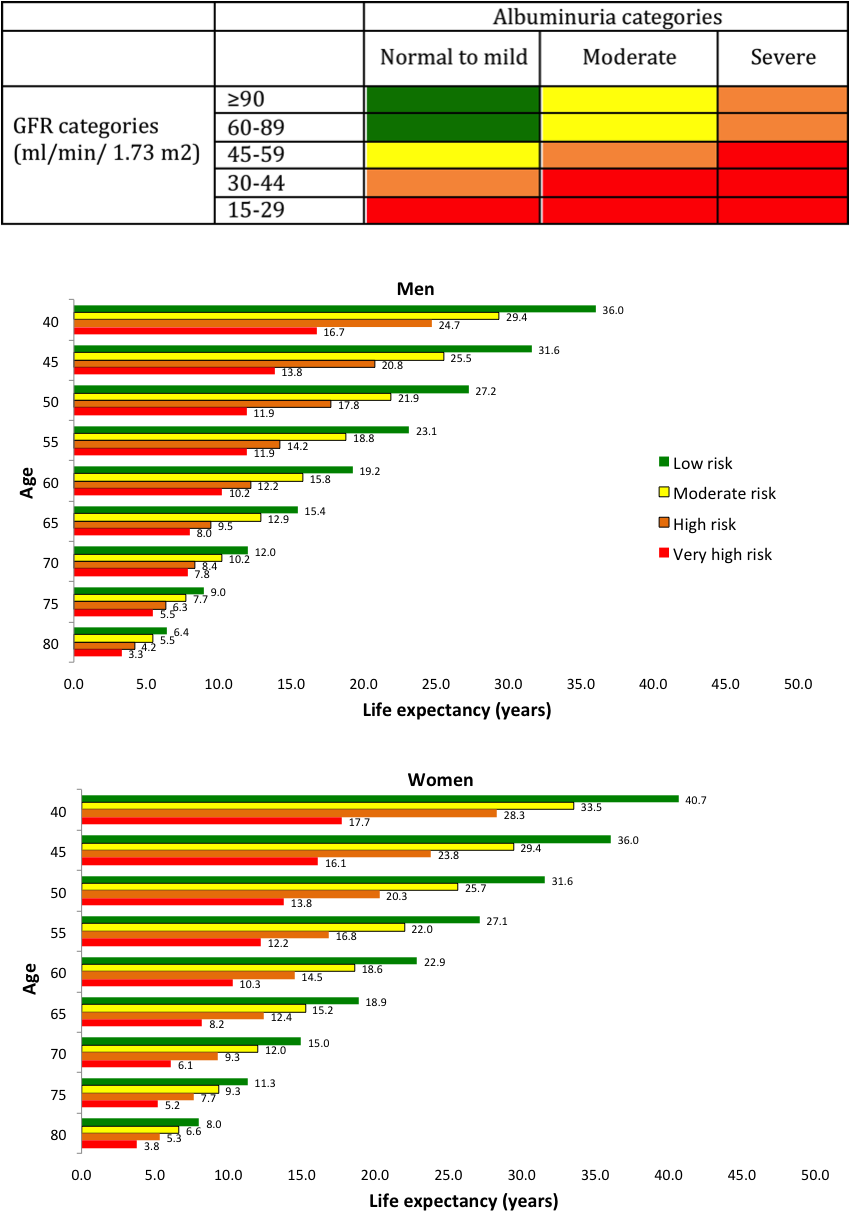


**Supplementary Figure 2**


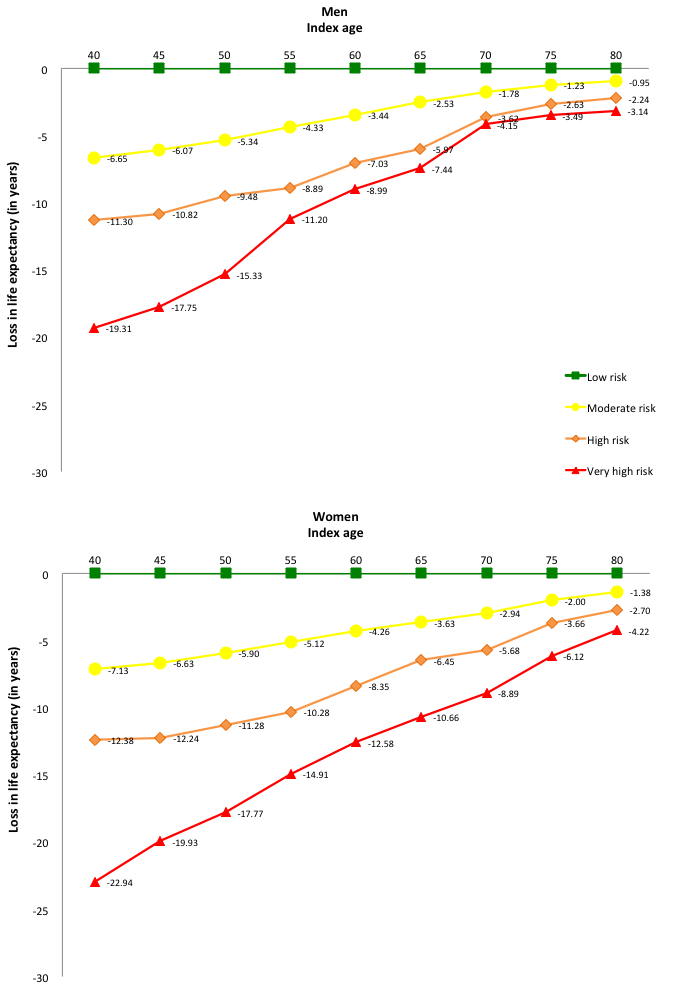

Supplement: Additional file 1: — Life expectancy among men and women at different index ages across severity of chronic kidney disease. [file 40697_2014_33_MOESM1_ESM.doc]
